# Par14 interacts with the androgen receptor, augmenting both its transcriptional activity and prostate cancer proliferation

**DOI:** 10.1002/cam4.5587

**Published:** 2022-12-30

**Authors:** Miki Naito, Kenichiro Ikeda, Shunya Aoyama, Mayu Kanamoto, Yasuyuki Akasaka, Yuri Kido, Mikako Nakanishi, Machi Kanna, Takeshi Yamamotoya, Akio Matsubara, Nobuyuki Hinata, Tomoichiro Asano, Yusuke Nakatsu

**Affiliations:** ^1^ Department of Medical Chemistry, Graduate School of Biomedical and Health Sciences Hiroshima University Hiroshima Hiroshima Japan; ^2^ Department of Urology, Graduate School of Biomedical and Health Sciences Hiroshima University Hiroshima Hiroshima Japan; ^3^ Department of Urology Hiroshima General Hospital Hatsukaichi Hiroshima Japan

**Keywords:** androgen receptor, androgen signaling, cell proliferation, Par14, prostate cancer

## Abstract

**Background:**

Prostate cancer (PCa) is a major cause of cancer morbidity and mortality for men globally, and androgen signaling clearly drives its onset and progression. Androgen receptor (AR) regulation is complex and remains elusive, despite several studies tackling these issues. Therefore, elucidating the mechanism(s) underlying AR regulation is a potentially promising approach to suppressing PCa.

**Methods:**

We report that Par14, one isoform of the prolyl isomerases homologous to Pin1, is a critical regulator of AR transcriptional activity and is essential for PCa cell growth.

**Results:**

Par14 was shown to be overexpressed in PCa, based on analyses of deposited data. Importantly, overexpression of Par14 significantly enhanced androgen‐sensitive LNCap cell growth. In contrast, silencing of Par14 dramatically decreased cell growth in LNCap cells by causing cell cycle arrest. Mechanistically, silencing of the Par14 gene dramatically induced cyclin‐dependent kinase inhibitor p21 at both the mRNA and the protein level through modulating the localization of p53. In addition, suppression of Par14 in LNCap cells was shown to downregulate the expressions of androgen response genes, at both the mRNA and the protein level, induced by dihydrotestosterone. Par14 was shown to directly associate with AR in nuclei via its DNA‐binding domain and augment AR transcriptional activity.

**Conclusion:**

Thus, Par14 plays a critical role in PCa progression, and its enhancing effects on AR signaling are likely to be involved in the underlying molecular mechanisms. These findings suggest Par14 to be a promising therapeutic target for PCa.

## Graphical abstract

Prolyl isomerase Par14 directly associates with the androgen receptor. Par14 is mandatory for cell proliferation and its transcriptional activity.

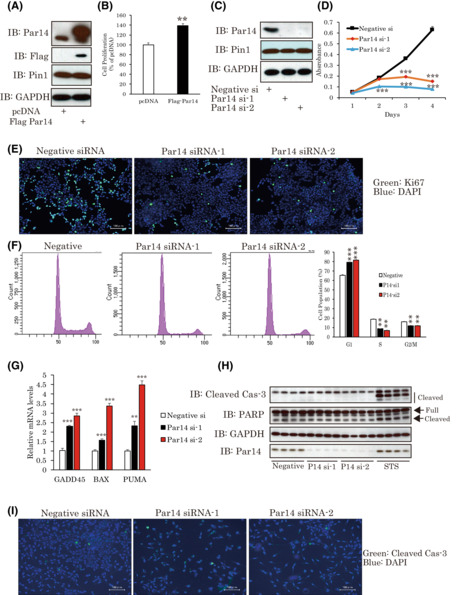

## 1 INTRODUCTION

Recently, prostate cancer (PCa) has been increasing worldwide, likely due to dietary changes, prolonged life span and perhaps other as yet unknown factors.^1^, ^2^, ^3^ Accordingly, the death toll from PCa has also obviously risen and much attention has been paid to developing early detection, prognostic evaluation and new therapeutic methods.^4^, ^5^, ^6^ The androgen hormone is well known to govern the development of PCa, via a mechanism similar to that of its effect on normal growth of prostate gland tissue through its nuclear receptor.^7^, ^8^ Thus, therapy involving androgen ablation or suppression of its receptor signaling exerts efficacy against PCa in most cases, although such androgen‐sensitive PCa tumors eventually change into castration‐resistant prostate cancer (CRPC).^9^, ^10^, ^11^

Androgen receptor (AR) signaling is classically evoked through multiple steps by androgens, such as dihydrotestosterone (DHT), and many co‐factors have been shown to participate in these processes.^8^, ^12^, ^13^ In the low androgen state, AR associates with heat shock protein 90 (HSP90) and mainly exists in the cytosol. DHT stimulation drives the conformational changes of AR and this leads to detachment from HSP90. Subsequently, AR translocates to the nucleus in the form of a homodimer and binds to AR response elements in the genome. AR functions are also controlled by interacting with a variety of co‐factors, such as Creb‐binding protein, Yes‐associated protein and Forkhead box A1.^14^, ^15^, ^16^ In addition, peptidyl prolyl cis/trans isomerase 1 (Pin1) has been shown to associate with the N‐terminal domain of AR, leading to AR transactivation.^17^

Prolyl isomerases are unique enzymes which interact with and modify their target proteins by isomerizing proline residues.^18^, ^19^, ^20^ Currently, prolyl isomerases are divided into three families, FK506 binding protein (FKBP), Cyclophilin and Parvulin.^21^, ^22^ The FKBP and the Cyclophilin families are both well known as immunophilins, as they were identified as target proteins of immunosuppressive agents.^23^ On the other hand, the Parvulin family consists of Pin1 and Par14 (also referred to as Pin4).^20^ Pin1 correlates closely with the exacerbation of cancers and is, in fact, overexpressed in a wide variety of malignancies, such as prostate, ovarian and pancreatic cancers.^24^, ^25^, ^26^ In contrast to numerous reports unraveling important physiological as well as pathogenic functions of Pin1, the role of another Parvulin isoform, Par14, remains poorly understood.

In this study, we began by showing the high expression level of Par14 in PCa, and then demonstrated that Par14 is essential for the proliferation of PCa. Moreover, Par14 interacts with AR and enhances its transcriptional activity. These findings suggest Par14 to be an attractive target for the discovery of drugs targeting PCa.

## 2 MATERIALS AND METHODS

### 2.1 Cell culture

LNCaP cells were purchased from RIKEN. All cells were routinely cultured in RPMI1640 (Life Technologies) containing 10% fetal calf serum, glutamine, NaHCO_3_ and antibiotics. They were maintained in a humidified atmosphere of 95% air and 5% CO_2_ at 37°C. For DHT stimulation, LNCap cells were incubated in the medium supplemented with 10% charcoal‐stripped serum (CSS) overnight, and were then exposed to 10 nM DHT for the indicated times. DHT was purchased from Selleck Chemicals.

### 2.2 Analysis of deposited data

The expressions were compared between normal and tumor tissues based on TNM plot data (https://tnmplot.com/analysis/). This platform includes a dataset from GTex, TCGA and TARGET.^27^ Survival rates were calculated by Gene Expression Profiling interactive analysis (GEPIA, http://gepia.cancer‐pku.cn/).

### 2.3 Plasmid transfection and siRNA treatment

Plasmid transfection was performed by using X‐treme reagent (Roche). The plasmid and X‐treme were both placed in Opti‐MEM to allow formation of the complex for 15 min. Subsequently, these complexes were added to the cells.

For Par14 knockdown, siRNA treatment was conducted by the reverse‐transfection method. Briefly, all stars Negative or Par14 siRNA was mixed with RNAiMAX in Opti‐MEM for 15 min. The mixture was applied to each well, and the cells were then seeded. The following siRNA sequences were used.

Par14 si‐1: CCGCACAGTATAGTGAAGATA

Par14 si‐2: GGCCGCACAGTATAGTGAAGATAAA

### 2.4 Cell proliferation and migration assay

Cell proliferations were examined employing WST‐1 reagent (Takara Bio). Each cell was treated with Par14 plasmid or siRNAs. After 5 days, WST‐1 reagent was added to fresh medium and the cells were incubated for 3 h at 37°C. The absorbance of the supernatant was measured at 450 nm employing a Varioscan (Thermo Fisher Scientific).

### 2.5 Plasmid

The human Par14 gene was inserted into pcDNA3.1 (‐) with a Flag tag sequence in the N‐terminal. Flag‐M4‐AR was a generous gift from Steven Balk (Addgene plasmid #171240; http://n2t.net/addgene:171240; RRID: Addgene_171240). AR deletion mutants were inserted into a myc‐TEV‐Flag‐pcDNA vector. PSA5.8k‐Luc reporter vector, which includes the 5.8 kb PSA enhancer/promoter region, was kindly provided by Dr. A. Mizokami (Kanazawa University).^28^, ^29^ This region contains the enhancer sequence of the candidate AR binding site (GGAACATATTGTATC).

### 2.6 Proximity ligation assay

A proximity ligation assay (PLA) was conducted, as described in a previous report.^30^ Briefly, LNCap cells were fixed with 10% formalin and then permeabilized with 0.1% Triton. Subsequently, after they had been blocked with 3% goat serum, two primary antibodies were added and the slide chambers were incubated at 4°C overnight. The next day, cells were incubated with PLA probes which had been conjugated with anti‐mouse or anti‐rabbit secondary antibodies for 1 h at 37°C. The cells were then reacted with ligase to form the circular DNA strands for 30 min at 37°C. Finally, rolling circle amplification and labeling with fluorescent probes were conducted for 100 min at 37°C. During each process, slide chambers were washed with phosphate buffered saline (PBS) three times. Fluorescent signals were observed and recorded under a BZ‐8000 microscope (Keyence).

### 2.7 Flow cytometry

LNCap cells were treated with siRNAs. After 3 days, the cells were collected. Subsequently, they were fixed with ice‐cold ethanol for 2 h at 4°C. After washing with PBS, the cells were mixed with propidium iodide/RNase staining buffer (BD Biosciences) for 15 min. Samples were washed again and analysis of the cell cycle was carried out employing the LSR Fortessa X‐20 (BD Biosciences).

### 2.8 Western blotting

Western blotting was performed, as described in previous reports.^26^, ^30^ Briefly, proteins were separated by sodium dodecyl sulphate‐polyacrylamide gel electrophoresis and then transferred onto polyvinylidene fluoride membranes. After blocking with 3% skim milk or bovine serum albumin for 1 h, membranes were incubated with primary antibodies for 1 h. They were then washed with PBS containing 0.1% Tween20 three times, and finally reacted with secondary antibodies (1:4000) for 1 h. Membranes were washed again and proteins were detected using a Clarity Western ECL system (Bio‐rad Laboratories).

Primary antibodies were diluted as follows: AR (Cell Signaling 5153; 1:4000), GAPDH (Proteintech 60004‐1‐lg; 1:10,000), PSA (Cell Signaling 5365; 1:3000), NKX3.1 (Cell Signaling 92998; 1:3000), KLK2 (Proteintech 10812‐1‐AP; 1:3000), p21 (Cell Signaling 2947; 1:3000), Par14 (Proteintech 15789‐1‐AP; 1:3000), and Flag antibody (Sigma F1804; 1:4000). Par14 antibody was also raised by the injection of GST‐Par14 into rabbits. Primary and secondary antibodies were diluted in 3% skim milk or Can Get Signal (Toyobo).

### 2.9 Real time PCR

Total RNA was extracted using a NucleoSpin RNA kit, according to the manufacturer's protocol. After RNA concentrations had been measured using Nanodrop, the same amount of cDNA was then created using reverse‐transcriptase with a verso cDNA kit. Then, real time PCR was conducted employing SYBR Green and each specific primer. Heatmap was created by Heatmapper (http://www.heatmapper.ca/). The primers used were as follows.

hPSA (KLK3) F: atgacgtgtgtgcgcaagtt R: atggttcactgccccatgac

hKLK2 F: aggcacacacacagcaagga R: gggggacctgaacaaacctc

hNKX3.1 F: cttccaaggcttccccaaac R: agctccgaggagagctgctt

hPUMA F: tggactcagcatcggaaggt R: tgaaggagcaccgagaggag

hBAX F: gcgtccaccaagaagctgag R: gccttgagcaccagtttgct

hGADD45 F: atggataaggtgggggatgc R: gcctggatcagggtgaagtg

h p21 F: tcaaatcgtccagcgacctt R: tggaaggtgtttggggtcag

h p27 F: gggcaagtacgagtggcaag R: gtccaccaaatgcgtgtcct

hPar14 F: aaggggcttgtacggcaact R: actgcattgccaccaccttt

hGAPDH F: ggtgaaggtcggagtcaacg R: agggatctcgctcctggaag

### 2.10 Luciferase assay

LNCaP cells were cultivated on a 48‐well plate overnight, and then transfected with both PSA‐Luc and TK‐rluc vector, using X‐treme reagent. One day thereafter, the cell medium was switched to medium containing CSS. The next day, the cells were stimulated with DHT for 24 h, and the luciferase assay was performed using a Dual‐Glo Luciferase assay kit (Promega), according to the manufacturer's protocol.

### 2.11 Immunoprecipitation

The indicated plasmids were transfected into LNCap cells. After 2 days, the cells were solubilized by lysis buffer containing Tris–HCl, NaCl, ethylenediaminetetraacetic acid, 0.1% Triton, phenylmethylsulfonyl fluoride, NaF and orthovanadate. After centrifugation, the cell lysates were transferred into new tubes and flag beads were added. Samples were rotated for 2 h at 4°C, and the beads were then washed with lysis buffer four times. Finally, 2DB was added to each tube and the samples were heated at 95°C for 10 min.

### 2.12 Immunostaining

Cells were cultivated in an 8‐well chamber. They were fixed with 10% formalin for 10 min, then for 5 min in 0.1% Triton. For blocking, 3% goat serum was added to each well for 30 min. Thereafter, cells were reacted with primary antibody (1:300) at 4°C overnight. Subsequently, the cells were mixed with Alexa488 (1:400) at room temperature for 1 h. Finally, cells on the slide were mounted with 4′,6‐diamidino‐2‐phenylindole and observed by BZ8000. After each process, the chamber slide was washed with PBS three times.

### 2.13 Statistical analysis

All data are presented as means + standard error of mean. Statistical significance was calculated employing the BellCurve for Excel.

**FIGURE 1:**
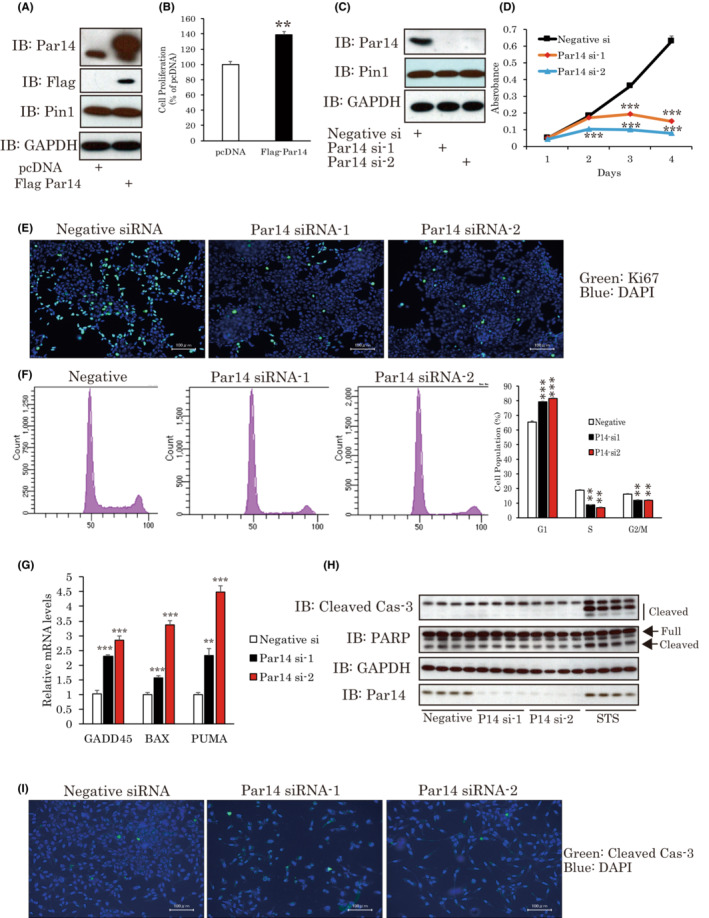
Par14 is significantly involved in the proliferation of PCa. (A, B) Par14 overexpression upregulated the cell growth of LNCap. Flag‐Par14 was transfected into LNCap cells. After 5 days, WST‐1 was conducted (*n* = 4). (C, D) Par14 knockdown dramatically diminished cell proliferation. LNCap cells were treated with Par14 siRNAs for 4 days. The resulting cell growth was then measured (*n* = 4). (E) Ki67 staining. LNCap cells were treated with Par14 siRNAs. After 72 h, the cells were stained with Ki67 antibody. Representative images are shown. (F) Par14 genes in LNCap cells were silenced, and cell cycle analysis was then carried out by PI staining and FACS (*n* = 3). (G) Cells were treated with siRNAs for 2 days. Then, the expressions of apoptotic genes were measured by real time PCR. (H, I) The detections of cleaved caspase‐3 and PARP. Two days after siRNA treatments, cleaved caspase‐3 or PARP was detected by immunostaining (G) or western blotting (H). Staurosporine (STS) is used as a positive control of apoptosis inducer. **p < 0.01 versus pcDNA or Negative siRNA, ***p < 0,001 versus Negative siRNA. PCa, prostate cancer.

**FIGURE 2:**
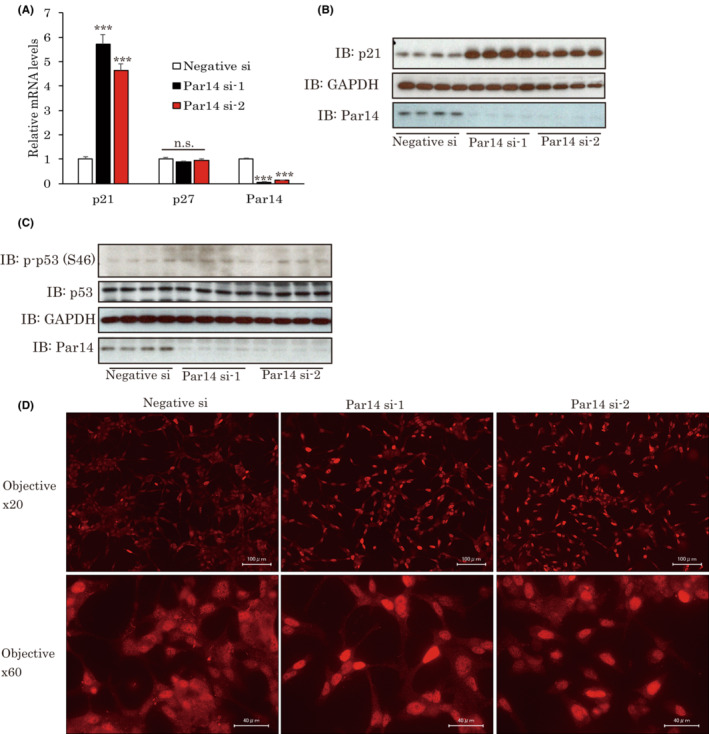
Par14 upregulates CDK inhibitor p21 by modulating the localization of p53. (A) Par14 knockdown upregulates p21 expression. LNCap cells were treated with siRNA for 2 days. Then, the expressions of CDK inhibitors were examined by real time PCR (*n* = 4). (B) Two days after the siRNA treatments, p21 protein levels were investigated by western blotting. (C) Protein levels of p53 were measured after siRNA treatments. (D) Par14 is involved in the localization of p53. LNCap cells were treated with siRNAs for 2 days. Then, immunostaining was performed employing the p53 antibody.

**FIGURE 3:**
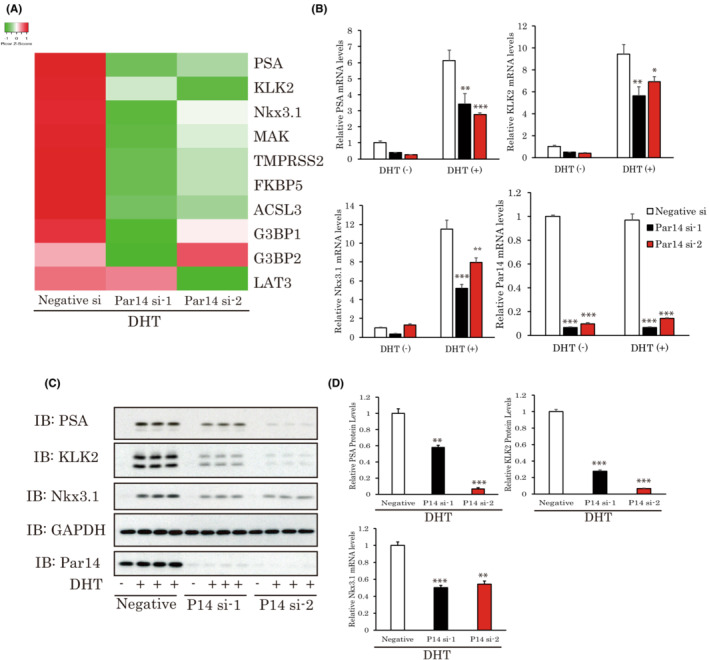
Silencing of Par14 attenuates the expressions of androgen receptor response genes. (A) Changes of androgen target genes in response to Par14 knockdown are shown as a heatmap. (B, C) The expressions of DHT‐induced genes were decreased by Par14 siRNA treatments. LNCap cells were treated with Par14 siRNAs for 2 days. The cells were then stimulated with 10 nM DHT for 8 h (A) or 24 h (B) (*n* = 4). (D) The band intensities of DHT‐treated samples were quantified. *p < 0.05, **p < 0.01, ***p < 0,001 versus Negative siRNA. DHT, dihydrotestosterone.

Student's‐*t* test was used for the analysis of WST‐1, flow cytometry, real time PCR (Figures 1 and 2) and western blotting results. Tukey–Kramer‐one way analysis of variance was also adopted for real time PCR (Figure 3) and luciferase assay data. The log‐rank test was performed to analyze the Kaplan–Meier curves. A value of *p* less than 0.05 was considered to indicate a statistically significant difference.

## 3 RESULTS

### 3.1 Like Pin1, Par14 is overexpressed in PCas

Although both Pin1 and Par14 belong to the Parvulin family, the WW domain in the N‐terminal of Pin1 is replaced with a Basic domain in Par14, which accounts for the difference in their binding motifs (Figure S1A). As Pin1 expression is well known to be upregulated in malignant tissues including PCa, we examined whether Par14 expression might also be elevated in PCa. For this purpose, we accessed the web tool TNM plot (https://tnmplot.com/analysis/) which pools the previously deposited RNA‐seq data.^27^ In this analysis, we found Par14, like Pin1, to be overexpressed in prostate adenocarcinoma (Figure S1B).

The survival rate of patients with PCa was also investigated using gene expression profiling interactive analysis (GEPIA: http://gepia.cancer‐pku.cn/). The patients with PCa expressing high levels of Par14 had shorter overall survival (OS) periods as well as disease‐free survival (DFS) periods than those expressing low levels of Par14 (Figure S1C). However, the difference was only statistically significant for the DFS periods, i.e., not for OS periods. Survival rates in PCa patients are generally high, and recent advances in therapy have further prolonged life expectancy. Accordingly, Par14 expression levels might not correlate with OS. However, the relationship of Par14 with DFS may indicate that Par14 contributes to unfavorable outcomes, such as recurrences. In other words, Par14 might affect quality of life in PCa patients.

### 3.2 Par14 silencing attenuates PCa cell proliferation

To examine whether Par14 is required for PCa proliferation, we utilized LNCap cells which have the AR and are sensitive to androgen stimulation, as androgen depletion therapy is still the gold standard and, also, we were interested in examining the effects of Par14 on AR signaling.

In LNCap cells transiently overexpressing FLAG‐tagged Par14, we found that growth was slightly, but significantly, enhanced (Figure 1A,B). However, two different Par14 siRNA treatments dramatically downregulated its expression and blocked cell growth (Figure 1C,D). The Par14 expression changes exerted no effects on Pin1 protein levels (Figure 1A,C).

We investigated whether Par14 affected cell division or apoptosis in LNCap cells. Ki‐67 staining of cells revealed Par14 knockdown to impair cell proliferation (Figure 1E). To further confirm the necessity of Par14 for cell proliferation, cell cycle analysis by flow cytometry was carried out. Compared to control cells, in Par14 knockdown cultures, the populations of both G2 and S phase cells were downregulated, while the proportion in G1 phase was upregulated (Figure 1F).

To examine whether apoptosis contributes to the suppression of cell proliferation induced by Par14 knockdown, we assessed changes in apoptosis related genes. Apoptotic genes, such as GADD45, PUMA and BAX, were significantly upregulated by Par14 knockdown in the cells (Figure 1G). However, unexpectedly, neither cleaved caspase‐3 nor the cleavage of PARP, which reflects the occurrence of apoptosis, was detected in Par14 siRNA‐treated cells (Figure 1H,I), suggesting that Par14 promotes cell growth mainly by enhancing the cell cycle, rather than by preventing apoptosis. Taken together, these observations suggest that Par14 plays a critical role in the proliferation of PCa cells.

### 3.3 Par14 is involved in the regulation of CDK inhibitor p21 expressions

The apoptotic genes described above are representative factors induced by p53. It is well known that p53 has the ability to upregulate CDK inhibitor p21, in turn leading to cell cycle arrest. Accordingly, we investigated whether Par14 knockdown affects p21 expressions in LNCap cells. The silencing of Par14 elevated p21 mRNA expressions, while p27, which is an another important CDK inhibitor, was unaffected (Figure 2A). Consistent with the changes in mRNA, p21 protein levels were also dramatically upregulated by Par14 knockdown (Figure 2B). Contrary to these changes, neither p53 expression nor phosphorylation levels were changed by treatments with siRNAs (Figure 2C). Next, we examined the localization of p53 in Par14 knockdown cells. When LNCap cells were treated with negative siRNA, p53 diffused into both the cytosol and the nucleus. However, treatments with Par14 siRNAs enhanced p53 accumulation in the nucleus (Figure 2D).

Taken together, our observations indicate that Par14 knockdown affects the localization of p53, and that, consequently, cell cycle arrest can be induced by upregulating p21 expressions.

### 3.4 Par14 is committed to androgen‐induced responses

As a next step, we examined whether Par14 participates in regulating androgen response genes. For this purpose, we measured the expressions of androgen target genes in Par14 siRNA‐treated LNCap cells by real time PCR. Silencing Par14 by applying siRNAs significantly downregulated the expressions of these genes (Figure 3A), and the results of three representative genes are shown in Figure 3B. We also evaluated the influences of Par14 on their protein expressions. Consistent with mRNA levels, the addition of DHT dramatically raised their protein levels, and the suppression of Par14 blunted these elevations (Figure 3C). Quantitative analysis revealed these suppressions to be statistically significant (Figure 3D).

These results strongly suggest Par14 to be involved in the regulation of androgen signaling.

### 3.5 Par14 interacts with AR

**FIGURE 4:**
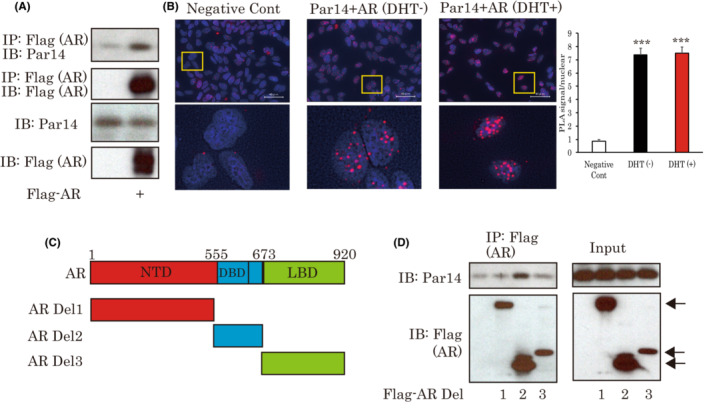
Par14 associates with AR through DNA‐binding domain. (A) The interaction between Par14 and AR was confirmed in LNCap cells. Flag‐AR was overexpressed in LNCap cells, and immunoprecipitation was then performed using Flag beads. (B) The endogenous association between Par14 and AR demonstrated by the proximity ligation assay method. (C) Constructs of AR deletion mutants (D) Identification of the AR domain interacting with Par14. The indicated plasmids were transfected into LNCap cells. Two days later, the cells were immunoprecipitated with Flag beads. Arrows indicate AR del1, del2 or del3. AR, androgen receptor.

As Par14 was found to be involved in DHT‐induced responses, we examined whether Par14 might be associated with AR. When Flag‐AR was overexpressed in LNCap cells, the interaction between AR and Par14 was confirmed (Figure 4A). For the detection of endogenous binding, we carried out a PLA. Employing this method, we detected the endogenous association between Par14 and AR mainly in the nucleus (Figure 4B). Next, we endeavored to identify the region of AR responsible for binding with Par14. AR has three main domains which are NTD, DBD and LBD. For the activation of AR, the binding of ligands to LBD is essential, and DBD is responsible for binding on DNA. In addition, Pin1 reportedly enhances AR functions via an association with NTD. Thus, to elucidate the possible roles of Par14 in AR functions and/or the relationship with Pin1, identification of the AR domain which interacts with Par14 would provide useful information.

Deletion mutants for each of these domains were created and whether they retained the ability to bind with Par14 was investigated (Figure 4C). We found that Par14 bound to the AR with the DBD domain (Figure 4D).

### 3.6 Par14 enhances AR transcriptional activity

**FIGURE 5:**
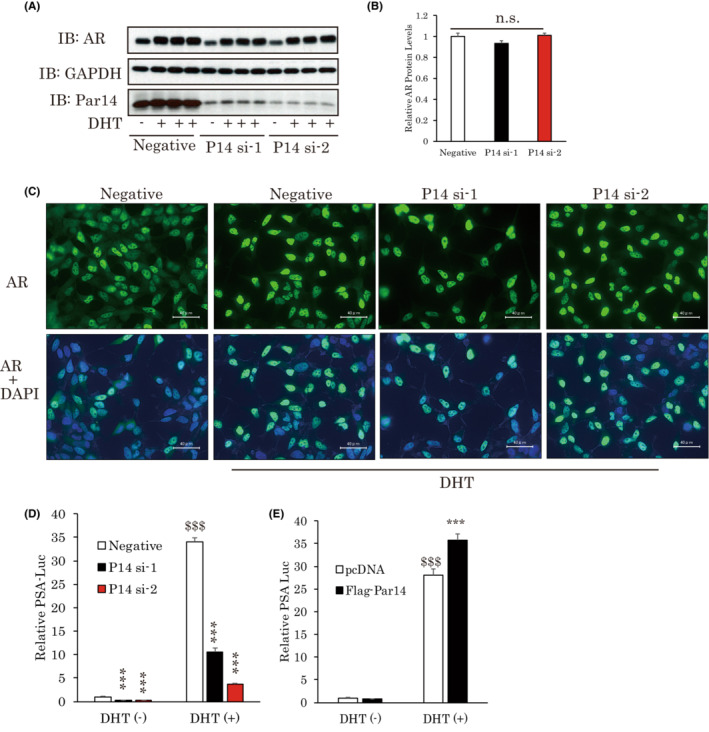
Par14 enhances AR transcriptional activity without affecting either the expression or the translocation of AR. (A–C) AR expression or translocation is unaffected by Par14 knockdown. LNCap cells were treated with Par14 siRNA. Subsequently, the cells were stimulated with DHT for 24 h (A) or 8 h (C). (D, E) Par14 promotes AR transcriptional activity. After treatment with siRNAs, luciferase vectors were delivered into LNCap cells, followed by incubation, with DHT added to the medium, for 24 h (*n* = 4). For Par14 overexpression, Flag‐Par14 was transfected with the PSA‐Luc vector in cells (*n* = 4–5). ^$$$^ *p* < 0.001 versus Cont or Negative DHT (−), ***p < 0.001 versus Negative DHT (+). AR, androgen receptor; DHT, dihydrotestosterone.

To clarify the regulation of AR by Par14, we began by examining the impacts of Par14 on AR expression levels. A number of studies have shown AR protein to be stabilized by DHT treatment.^17^, ^31^, ^32^ Consistently with previous reports, the addition of DHT in LNCap cells upregulated AR protein levels. However, these elevations were unaffected by the suppression of Par14 (Figure 5A,B). Androgen stimulation also enables AR to translocate from the cytosol to the nucleus. Although we confirmed that AR translocation occurred in response to DHT addition, AR was mainly observed in the nucleus even when Par14 was silenced (Figure 5C).

Finally, we considered the possibility of Par14 facilitating AR transcriptional activity. We used the PSA‐Luc vector which includes the 5.8k enhancer/promoter region of PSA. Interestingly, the suppression of Par14 strongly suppressed AR transcriptional activity (Figure 5D), while Par14 overexpression accelerated it (Figure 5E).

These results indicate that Par14 enhances AR activity without affecting either its stability or translocation.

## 4 DISCUSSION

Par14 and Pin1 belong to the Parvulin family of prolyl isomerases. While their isomerase domains on the C‐terminal side are highly conserved, their N‐terminal domains are not homologous. This difference in N‐terminal domains (WW domain of Pin1 and Basic domain of Par14) (Figure S1A) accounts for the difference in binding motifs and the resultant target proteins between Pin1 and Par14. Nevertheless, the reported evidence that Pin1 suppression induces increased expression of Par14 may suggest some redundancy in the roles of Pin1 and Par14.^33^ Pin1 reportedly binds to more than 100 proteins via recognition of their phospho‐Ser or –Thr/Pro containing motifs and is involved in several important diseases.^20^, ^22^, ^34^ They include cancers, Alzheimer disease, metabolic disorders such as obesity and hepatosteatosis, as well as inflammatory and fibrotic diseases.^22^, ^35^, ^36^, ^37^ Notably, a high expression level of Pin1 is involved in the exacerbation of a variety of cancers, induced by regulating many proteins related to cell proliferation, cell cycle apoptosis and so on.^34^, ^35^ Compared with Pin1, fewer reports have been published regarding the roles of Par14, and no evidence of a relationship with cancer has yet been obtained.

In this study, we began by demonstrating Par14 to be overexpressed in PCa as compared with normal tissues, based on analyses of deposited data, and this overexpression was more evident than that of Pin1 (Figure S1B). Importantly, siRNA‐mediated suppression of Par14 dramatically decreased cell growth and caused cell cycle arrest in androgen‐sensitive LNCaP. As a mechanism, we found that silencing of the Par14 gene dramatically induced CDK inhibitor p21 by modulating the localization of p53. Previously, p21 expressions were reported to be indirectly downregulated by androgen. The Imaizumi group revealed that androgen‐induced AlbZIP suppresses p21 expressions via impairment of the functions of transcriptional factor OASIS.^38^ In addition, another team demonstrated that androgen stimulation promotes translocation of p53 from the nucleus to the cytosol.^39^ Considering our observation that Par14 enhances the expressions of genes downstream from AR, Par14 might increase PCa cell growth through the regulation of AR signaling, and regulations of p53 by Par14 may, at least partially, contribute to the proliferation of this cell line.

A direct association between Par14 and AR was demonstrated. As to PCa, there are studies indicating Pin1 to be a critical regulator for both PCa proliferation and AR signaling.^17^, ^40^ AR consists of three main domains, i.e., NTD, DBD and LBD. Interestingly, the AR binding sites differ between Pin1 and Par14. Pin1 was shown to associate with NTD and our results revealed DBD to be a binding area for Par14.^17^ These observations suggest that two enzymes might act in a coordinated fashion and thereby lead to the full activation of AR. As AR activation requires ligand binding, a major factor in androgen deprivation therapy (ADT) for PCa is androgen antagonism, and LBD is one of the main target domains for drug discovery.

However, as is well known, ADT eventually leads to ligand‐independent AR activation, so‐called CRPC. This phenomenon is partially explained by the manifestation of AR‐V7 which is LBD lacking a receptor.^41^, ^42^ Therefore, inhibiting AR, independently of LBD, might potently suppress PCa. Par14 interacts with DBD and can elevate AR transcriptional activity. Although no inhibitors of Par14 have yet been identified, their development as new anticancer drugs merits exploration.

We cannot exclude the possibility that Par14 promotes growth through an AR‐independent pathway, in addition to AR‐dependent signaling. Par14 is known to be expressed in both the nucleus and the cytosol according to the Human Protein Atlas database. We assume that Par14, like Pin1, interacts with a number of substrates to modulate cellular functions. In fact, both Pin1 and Par14 associate with insulin receptor substrate‐1 (IRS‐1), enhancing its insulin‐induced phosphorylation.^43^ We speculate that Par14 functions by interacting with multiple as‐yet‐unidentified signal transduction or transcriptional factors, similar to the actions of Pin1, and indeed, BioGrid (https://thebiogrid.org/111320/table/homo‐sapiens/pin4.html), which is a database of protein interactions, showed the presence of numerous Par14 binding partners. These findings raise the possibility that Par14 has divergent roles in cell growth and other physiological functions.

As to the limitations of this study, first, we used only single AR‐positive cells, because we cannot obtain others from cell banks in Japan. As is well known, androgen ablation therapy causes the development of CRPC which was, at least partially, triggered by the expression of AR‐V7. Cancer tissues are of course heterogonous, and Par14 might have distinct roles, depending on cell types. Accordingly, future studies are required to fully clarify the roles of Par14 in the functions of AR and its splicing variants, such as AR‐V7. In addition, unfortunately, no Par14 inhibitors have as yet been developed and we thus cannot examine their effects on the in vivo growth of PCa. Creating Par14 inhibitors and conducting in vivo trials might facilitate understanding the effects of Par14 on the survival rate and/or proliferation under physiological conditions.

In conclusion, the findings of our current study reveal that Par14 plays a critical role in PCa progression, and its enhancing effect on AR signaling may well contribute to the underlying molecular mechanisms. Accordingly, Par14 is a key molecule for suppressing PCa and its inhibitors are promising targets for novel therapies.

## AUTHOR CONTRIBUTIONS

**Miki Naito:** Data curation (lead); investigation (lead). **Kenichiro Ikeda:** Data curation (supporting); investigation (supporting). **Shunya Aoyama:** Investigation (supporting). **Mayu Kanamoto:** Investigation (supporting). **Yasuyuki Akasaka:** Investigation (supporting). **Yuri Kido:** Investigation (supporting). **Mikako Nakanishi:** Investigation (supporting). **Machi Kanna:** Supervision (supporting). **Takeshi Yamamotoya:** Supervision (supporting). **Akio Matsubara:** Project administration (supporting); supervision (equal). **Nobuyuki Hinata:** Project administration (supporting); supervision (supporting); writing – original draft (supporting). **Tomoichiro Asano:** Conceptualization (supporting); funding acquisition (lead); project administration (equal); supervision (equal); validation (equal); writing – original draft (equal); writing – review and editing (equal). **Yusuke Nakatsu:** Conceptualization (lead); data curation (lead); funding acquisition (equal); investigation (lead); project administration (equal); supervision (equal); validation (equal); visualization (equal); writing – original draft (equal); writing – review and editing (equal).

## FUNDING INFORMATION

This work was partially supported by Grants‐in‐Aid for Scientific Research (C) (To Y.N. 19K08982, 22K08655) and (B) (To T.A. 20H03732). This research was also supported by the Nozomi H Foundation, Nagao Takeshi Orphan Disease Research Foundation and the Institute for Adult Diseases Asahi Life Foundation.

## CONFLICT OF INTEREST

The authors have no conflicts of interest.

## ETHICS STATEMENT

N/A.

## Supporting information


Figure S1.

## Data Availability

This manuscript does not include any unique or meta datasets. All data are deposited in the manuscript.
