# Supplementary material for: Par14 interacts with the androgen receptor, augmenting both its transcriptional activity and prostate cancer proliferation
Source: Cancer Med. 2022 Dec 30;12(7):8464–75. doi: 10.1002/cam4.5587 (PMC10134346; doi:10.1002/cam4.5587)

Supplementary Fig.1

(A) The structures of Pin1 and Par14 (B) Par14 expressions were compared between normal and tumor tissues using the TNMplot database (Normal n=204, Tumor n=498). Significant differences were detected by applying the Mann-Whitney U test. (C) Analysis of overall survival and disease-free survival periods of patients with PCa. These data were cited from GEPIA.


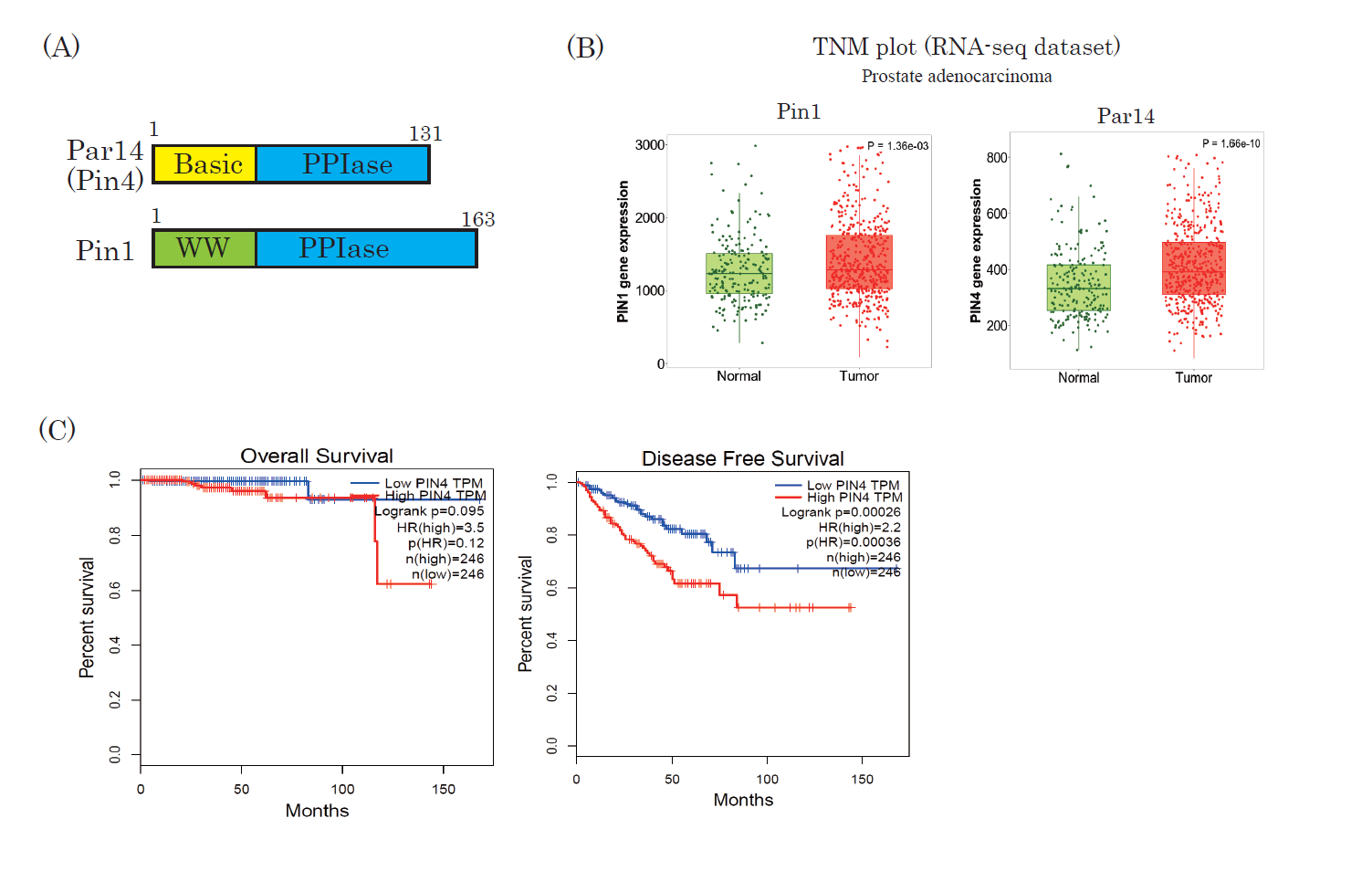

Supplement: Supplementary file 1 — Figure S1. [file CAM4-12-8464-s001.docx]
